# Legal Steps to Secure the Tobacco Supply Chain: A Case Study of Poland

**DOI:** 10.3390/ijerph17062055

**Published:** 2020-03-20

**Authors:** Łukasz Balwicki, Michal Stoklosa, Małgorzata Balwicka-Szczyrba, Jeffrey Drope

**Affiliations:** 1 Department of Public Health and Social Medicine, Medical University of Gdańsk, 42A Zwycięstwa St., Gdańsk 80-210, Poland; 2 American Cancer Society, 250 Williams St., Atlanta, GA 30303, USA; michal.stoklosa@cancer.org (M.S.); jeffrey.drope@cancer.org (J.D.); 3 Department of Commercial Law, University of Gdańsk, 6 Bazynskiego St., Gdańsk 80-309, Poland; malgorzata.balwicka@prawo.ug.edu.pl

**Keywords:** tobacco crops, tobacco supply chain, tobacco control, Poland

## Abstract

The threat of tobacco tax evasion and avoidance is the most commonly mentioned argument against tax hikes. Increasingly, the focus of legislators is on leaks in the tobacco crop supply chain, in which raw or cured tobacco that was never taxed finds its way to smokers. To study the process undertaken by Poland to secure the tobacco supply chain, we analyzed the 2013–2018 legislation around tobacco supply and interviewed a key stakeholder in the Government of Poland. We found that farmers and intermediary entities can trade tobacco only if registered with the government. Farmers are required to report the size of their fields and the weight of their crops to the state authorities. Each purchase within the supply chain is also reported by both the seller and the buyer for cross-validation. This has prevented manipulation within the system, while the mere threat of heavy fines related to an excise tax law violation and/or the administrative burden associated with becoming an excise tax payer (had the violation been prosecuted) has significantly contributed to securing the tobacco supply chain. The experience of Poland demonstrates that securing the tobacco supply chain is complicated but also a tractable problem. This case can be widely applicable to other countries.

## 1. Introduction

Tobacco taxes and mosquito nets share similarities. Both are strategies to protect people from a disease-spreading agent; both also work best without holes. Although keeping the tobacco supply chain sealed is crucial for the effectiveness of tobacco taxation, the task is difficult. Many countries around the world continue to struggle with tax avoidance and evasion, and this is almost always related to the effective protection of the tobacco supply chain.

With cigarette prices increasing, the problem is becoming ever more urgent. In Zambia, for example, nearly 40% of smokers now smoke roll-your-own cigarettes made mostly from cheap, untaxed loose tobacco which comes straight from the tobacco fields, with no clear government plan on how to address this problem [1]. In South Asia, tobacco that leaked from the official supply chain was used by illicit tobacco product manufacturers [2]. The European Commission is considering taking strong legislative measures against illicit trade in raw tobacco in the European Union (EU) with the next renewal of the EU’s tobacco tax directive [3]. Accordingly, to provide a pragmatic perspective on these issues, we present here a detailed description of the process undertaken by Poland to secure its tobacco supply chain, documenting its best practice approach to supply chain regulation and the obstacles encountered.

## 2. Materials and Methods 

To describe the legal steps to secure the tobacco supply chain in Poland, legislation around tobacco supply implemented from 2013 to 2018 was identified with the use of Legal Information System LEX (Wolters Kluwer Poland). We searched for legal acts relevant to the issue using the keyword phrase “tobacco taxation”. The main legal act is the Act of 6.12.2008 on excise duty, which was amended 49 times in the years 2013–2018. Acts were analyzed with the use of a formal-dogmatic method—a method typically used in legal analyses. Also, internet searches were performed to identify and analyze newspaper articles and correspondence between the government and members of parliament concerning tobacco taxation.

Additionally, an interview with Mr Bogdan Bednarski, Commissioner of the Expert of Customs and Tax Service, Department for Combating Economic Crime, Polish Ministry of Finance was conducted on 30 December 2018. 

## 3. Results

Efforts to secure the tobacco supply chain started when the manufacturers of smoking tobacco observed large declines in sales of their products. Until 2013, the only categories of tobacco and tobacco products subject to excise tax in Poland were cigarettes, cigars, cigarillos and smoking tobacco [4]. Even though Poland was, and still is, a tobacco-growing country [5], crops were not taxed, and both farmers and dealers could sell harvests to any person or company. Consequently, with the price difference between the taxed smoking tobacco and the untaxed cured—but still unmanufactured—tobacco, smokers were increasingly using the latter for cigarette rolling. It is estimated that, in 2012, as much as 70% of all roll-your-own cigarettes smoked in Poland were made from untaxed, cured tobacco, resulting in approximately PLN 1 billion (US$ 307 million) of lost government revenue annually [6].

The road toward eliminating these leaks was not easy. The government amended the excise tax law in December 2012 to close loopholes in the old bill. Starting in 2013, cured tobacco, defined as “dry tobacco, which is not yet a tobacco product”, became subject to excise tax [7]. The excise tax was waived if cured tobacco was sold to a tobacco manufacturer with a bonded warehouse or to a legitimate intermediary entity. Each of those entities was required to be registered in the official registry of Tobacco Intermediary Entities to trade raw tobacco without being subject to excise taxation. The rationale for continuing to permit intermediaries was to reduce the administrative burden on farmers. Being an excise tax payer can be burdensome in terms of both the time and costs associated with adherence to tax rules. With the new bill, the farmers were still free from the excise tax liability, provided they sold their crops to legitimate buyers within the official tobacco supply chain [7].

It soon became evident, however, that the new bill had its own loopholes. Because cured tobacco was defined by the new bill as ”dry tobacco”, the excise tax could still be circumvented if tobacco leaves were sold when moist. To avoid the tax, sellers were offering raw, uncured tobacco. Others were purchasing cured tobacco abroad, moisturizing it, and importing it to Poland for sales without the excise tax duty [8]. Moist tobacco leaves were sold in special packages to stabilize product moisture levels [9]. Store clients were offered shredding machines to cut the leaves after purchase [9]. Curing machines as well as machines to fill cigarette tubes with cut tobacco were also available [8]. 

It took several years for the government to secure the tobacco supply chain effectively. First, a new law was passed in December 2013, changing the cured tobacco definition and making it independent from the leaves’ moisture levels [10]. In fact, the new law defined cured tobacco as tobacco separated from the living plant before it becomes a tobacco product. A year later, retailers who offered automated cigarette tube filling were forced to remove the filling machines from their stores or to become official cigarette manufacturers [11]. Starting in 2016, new excise tax stamps were introduced. Those stamps are affixed to packages containing cured tobacco, allowing for the product to be tracked. Cured tobacco dealers were obliged to report both purchase and sales to the government and even place a down-payment towards future excise tax [11]. According to the Ministry of Finance, this effectively reduced the illegal practice of cured tobacco sales to unauthorized entities without excise tax [12]. Finally, a law passed in late 2017 required each tobacco farmer to register with the state authorities and to report the size of their tobacco field for the current year as well as their crop weight for previous years. The report must include not only the weight for the harvested crop, but also the weight of any destroyed and stored crops. Destruction of crops must be supervised by a government official [13]. In addition, entities purchasing tobacco crops must register the weight of purchased tobacco to allow customs and tax control services to cross-validate the information [13]. The information is securely stored in a digital database.

## 4. Discussion

The experience of Poland demonstrates that securing the tobacco supply chain is somewhat complicated but also manageable (see Box 1). Even though it has passed some effective laws and regulations, the government still does not adhere to all the best practices to control the tobacco supply chain listed in the Protocol to Eliminate Illicit Trade in Tobacco Products (the Protocol) [14]. For example, Poland registers tobacco farmers instead of licensing them, as recommended by the Protocol. With no licensing, the government has little power to control the number of tobacco growers by granting or revoking the right to grow tobacco. The supply chain could be further secured by including raw tobacco in the Excise Movement and Control System (EMCS)—a computerized system for monitoring the movement of excise goods under duty suspension in the European Union. However, the measures that are currently in place serve as a strong deterrent to would-be violators. The mere threat of heavy fines related to an excise tax law violation and/or the administrative burden associated with becoming an excise tax payer if a violation were prosecuted significantly secured the leaks in the tobacco supply chain, especially at its early stages. The cross-validation of information provided by different entities significantly reduces the possibility of circumventing and/or defeating the system, such as through bribery. The tracking and tracing system that is now becoming operative in the entire European Union will further secure the supply chain by implementing an electronic system of tracing tobacco product movement from the manufacturer to the last level before the first retail outlet. 

In Poland, the system of continental law is in force, and the basic source of law is the legal act. At the same time, the Constitution of the Republic of Poland introduces the principle of freedom of economic activity, which can only be restricted by a legal act. Thus, there is a need to adapt the law to changing social realities, as well as practices on the border of the law, by amending legal acts. While the Polish experience might not be replicable under all conditions, the basic steps the government is taking to secure the supply chain can be undertaken in most countries. Even in low-income tobacco-growing countries in Sub-Saharan Africa, there is sometimes an existing system of registering tobacco farmers. Moreover, investment in both licensing and movement-monitoring systems will almost certainly reduce leaks in the tobacco supply chain, leading to substantial increases in revenue. This, in turn, will ensure a significant return on investment.

Box 1 **Steps to secure the tobacco supply chain in Poland, 2013–2018.**

- Excise tax imposed on dry tobacco
- Tobacco crops to be sold only to registered intermediary entities
- Cured tobacco definition independent from the leaves’ moisture levels
- Retailers offering automated cigarette tube filling forced to remove the filling machines from their stores or to become official cigarette manufacturers
- Introduction of excise tax stamps for cured tobacco
- Cured tobacco dealers obliged to report both purchase and sales
- Introduction of tobacco farmers registry for cross-validation with dealer registry

## 5. Conclusions

The experience of Poland demonstrates that securing the tobacco supply chain is complicated but also manageable. We believe that this case is widely applicable to other countries.
